# Reliability of Ultrasonographic Assessment of Sternal Micromotions by Physiotherapists in Patients with Median Sternotomy

**DOI:** 10.3390/jcm14113770

**Published:** 2025-05-28

**Authors:** Gianluca Libiani, Ilaria Arcolin, Marco Guenzi, Giacomo Milani, Massimo Pistono, Stefano Corna, Marco Godi, Marica Giardini

**Affiliations:** 1 Division of Physical Medicine and Rehabilitation of Veruno Institute, Istituti Clinici Scientifici Maugeri IRCCS, Gattico-Veruno, 28013 Piedmont, Italy; gianluca.libiani@icsmaugeri.it (G.L.); stefano.corna@icsmaugeri.it (S.C.); marco.godi@icsmaugeri.it (M.G.); marica.giardini@icsmaugeri.it (M.G.); 2 Division of Cardiac Rehabilitation of Veruno Institute, Istituti Clinici Scientifici Maugeri IRCCS, Gattico-Veruno, 28013 Piedmont, Italy; marco.guenzi@icsmaugeri.it (M.G.); massimo.pistono@icsmaugeri.it (M.P.)

**Keywords:** reliability, ultrasound, ultrasonographic assessment, physiotherapist, sternotomy, rehabilitation

## Abstract

**Introduction:** Median sternotomy carries post-surgical risks like sternal instability, requiring careful monitoring. Ultrasonography provides a real-time, quantitative assessment of sternal micromovements and has emerged as a promising tool for clinical evaluation. However, its reliability for assessing sternal micromovements post-surgery remains unclear. This study evaluated the inter-rater, intra-rater, and test–retest reliability of ultrasound performed by physiotherapists. **Methods:** Ultrasound was used to measure the distance between sternal edges in sternotomized patients along the X-axis and Y-axis. Measurements were taken under a resting position, during cough, and in two supine-to-sitting postural transitions (one using a rotational modality and the other with an individual device). Real-time ultrasound imaging acquisition was followed by off-line data elaboration. Assessments were conducted by multiple physiotherapists after a brief training period. Reliability was determined using intraclass correlation coefficients (ICCs), along with the standard error of measurement (SEM) and minimum detectable change (MDC90). ICC values > 0.75 were classified as excellent. **Results:** A total of 33 subjects with median sternotomy were included (5 women, age 66 ± 7 years). All reliability measurements (24 total) were rated as excellent in each condition examined, with intra-rater ICCs exceeding 0.90, except for on the X-axis during the postural transition using the individual device for supine-to-sitting. SEM values ranged from 0.23 to 0.64 mm, while MDC90 values ranged from 0.54 to 1.50 mm. **Conclusions:** Ultrasound demonstrated excellent reliability for assessing sternal micromotions when performed by physiotherapists with brief training. Given its reliability, cost-effectiveness, and ease of use, ultrasound sternal micromotions assessment could be integrated into post-surgical rehabilitation to enhance patient care.

## 1. Introduction

Sternal instability, with an underestimated incidence of 1–8%, is the most common complication after median sternotomy, which remains the gold-standard incision in cardiac surgery [1,2]. This condition is characterized by non-physiologic movement between the two halves of the sternum after surgery. Patients with sternal instability often experience symptoms such as a clicking sensation, pain, and discomfort, which can significantly limit their ability to carry out daily activities [3].

Early detection, measurement, and monitoring of sternal instability are essential to determine the most appropriate treatment and enhance post-surgery patient care [4,5,6]. Physiotherapists could play a key role in this, given their expertise in prescribing and monitoring physical activity during the early stages of post-surgical recovery. Nowadays, the assessment of sternal instability is becoming increasingly more achievable, as new and emerging tools are gaining popularity in their practice [7].

To date, the sternal instability scale (SIS) is the most widely used tool for assessing sternal stability through physical examination. However, as it relies on the clinician’s palpatory skills, it may be susceptible to subjective errors. Despite this, its authors have reported the scale as reliable [8], although it still lacks the ability to provide quantitative data.

In recent years, ultrasound has been suggested as a novel, non-invasive, and cost-effective clinical assessment tool for physiotherapists for the real-time evaluation of the surgically divided sternum [9]. This technique could allow for the simple and easy measurement and quantification of sternal micromovements, even during dynamic tasks.

In the literature, ultrasound imaging has been reported to have good to excellent reliability in patients following median sternotomy, with intraclass correlation coefficients (ICCs) often reaching extremely high values [9,10,11]. However, the available studies are limited and present several methodological constraints. To begin with, they do not assess all components of reliability (i.e., intra-rater, inter-rater, and test–retest reliability). Test–retest reliability, in particular, is frequently omitted or inadequately evaluated due to insufficient operator washout time [9], which can often lead to confusion with intra-rater reliability [12]. Moreover, test–retest reliability is the most clinically relevant component, as it reflects the variability in repeating the measurement procedure by different operators over time, making its accurate assessment essential [13].

Furthermore, the majority of the available studies lack sample size calculations and the number of recruited subjects is often small [9,11].

In addition, the evaluation setting does not always reflect real clinical environments, thus lacking ecological validity. In fact, authors often adopt a series of strategies to facilitate data acquisition, such as marking the measurement reference point, and maintaining it consistently across all measurements [9].

In some cases, an apparatus is used to hold the ultrasound probe instead of a human hand, with the patient seated and their back supported [9]. While this setup enhances measurement stability, it eliminates the possibility of using ultrasound assessment in dynamic movement contexts. In other instances, inter-rater reliability is inevitably compromised when video sampling is performed by a single operator, with only the subsequent measurements conducted by two operators [11,14]. While these approaches standardize data collection, they simultaneously eliminate the natural variability inherent in the real-world clinical application.

In this context, the use of a highly reliable tool allows for a quantitative evaluation of sternal micromovements, helping create a better distinction between safe and unsafe movements. Currently, there is no consensus on the exact sternal precautions that should be prescribed to patients. Some evidence suggests that certain sternal precautions could be excessively limiting, reducing patients’ ability to move early and thus postponing their functional recovery [15]. Postural transitions, in particular the supine-to-sitting one, are performed daily by patients and could place excessive mechanical stress on the surgically divided sternum, hindering optimal bone healing. The literature has proposed two techniques for the supine-to-sitting transition: one using a rotational modality and the other involving a tied rope (individual device for supine-to-sitting, “IDSS”). The latter appears to be less stressful on the sternal wound, as it has been shown to cause less pain and perceived effort [16]. A study on the reliability of ultrasound in measuring micromovements during these postural transitions is essential for identifying the optimal technique for minimizing sternal stress in the activities of daily living.

The aim of this study was to evaluate the reliability of ultrasound in measuring sternal micromotion in a post-cardiac surgery population. Specifically, this study aimed to assess intra-rater, inter-rater, and test–retest reliability while measuring sternal micromotion in the supine resting position, during cough, and during two supine-to-sitting postural transition techniques.

## 2. Materials and Methods

### 2.1. Participants

This monocentric observational study was conducted between July 2024 and September 2024 at the Istituti Clinici Scientifici (ICS) Maugeri, Institute of Veruno (Gattico-Veruno, Italy). All patients, admitted to the Cardiac Rehabilitation department of the ICS Maugeri and who underwent surgery with median sternotomy with stainless steel wire closure under general anesthesia, were screened for inclusion during the first week of hospitalization. The sample included subjects who had undergone an elective or emergency cardiac surgical procedure via a median sternotomy over the age of 18 years, with normal cognitive functioning (maximum of 2 errors in the Short Portable Mental State Questionnaire [17]), had stable clinical parameters, and were able to get out of bed autonomously.

Patients who had other important comorbidities (i.e., orthopedic surgery < 6 months before, neurological disease), had wound problems that could impair their ability to undergo the ultrasound (i.e., wound dehiscence), or had undergone previous surgery with median sternotomy were excluded. In addition, the presence of medical devices that could limit movement (i.e., drainage) and the medical prescription of wearing a sternal support harness 24 h/day were also considered as exclusion criteria. Moreover, patients who could not have the ultrasound scan taken within three weeks from surgery were not included.

The eligible participants were informed about the study protocol and provided written informed consent to participate. The study was approved by the ICS Maugeri, Italy (approval number #2448 CE).

### 2.2. Assessment and Data Collection

All subjects were evaluated on the day of admission by a physiotherapist with at least ten years of experience in cardiac rehabilitation. Relevant baseline data from participant’s medical history and/or surgical procedure notes were collected from each patient’s clinical medical record. Age, sex, height, bodyweight, smoking history, comorbidities, bra size, and presence of macromastia in women (EU bra size > 80) were noted. In addition, supine respiratory rate at rest, which could show an abnormal breathing pattern that may have predisposed subjects to postoperative respiratory complications, and measurement of chest circumference in an upright position at the mamillar line with the use of a tape measure, were also recorded. Finally, prior to ultrasound testing, the SIS was administered to all patients. This physical examination test aimed to assess sternal stability in cardiac surgery and consisted of a 4-point scale, ranging from a grade 0 that corresponded to a clinically stable sternum with no detectable motion or separation of the sternal edges, to a grade 3 that corresponded to a completely separated sternum with marked increased motion or separation of the sternal edges [8].

Subsequently, participants were evaluated using ultrasound imaging, which involved two phases: real-time imaging acquisition and off-line imaging data elaboration. All raters had no previous experience in ultrasound imaging, but they had attended 10 h of ultrasound training [18], consisting of 3 h of theoretical lectures and 7 h of supervised clinical practice with a clinical sonographer.

### 2.3. Real-Time Ultrasound Imaging Acquisition

The imaging acquisition process started with the evaluation of sternal micromotion in a supine resting position and then during three functional activities (cough and postural change from the supine to the sitting position using a rotational technique and using the IDSS) by placing the ultrasound probe in the 4th intercostal space. The ultrasound probe was held with minimal pressure for the duration of each task to allow the acquisition of the optimal ultrasound clips.

The supine and cough assessments were performed with the patient’s arms by his side in the semi-Fowler’s position, lying on the bed with a trunk inclination of 30°. For the postural transitions, patients started from the semi-Fowler’s position and ended up in a sitting position on the bed with their feet on the floor [19].

One familiarization trial for each modality was allowed and standardized instructions were given for each task. In particular, regarding the two postural transition techniques, all patients were instructed as follows:

Rotational postural change [20]: (a) Bring your feet toward the edge of the bed; (b) roll onto your side; (c) lower your legs off the bed; and (d) push yourself into a sitting position using your arms, keeping them as close to your torso as possible.

Postural change using IDSS [16]: (a) Shift your legs toward the edge of the bed, positioning them diagonally; (b) grasp the IDSS with one hand, palm facing upward; (c) with the other hand, also palm up, hold the hand already gripping the IDSS; (d) pull on the IDSS by bending your elbows while simultaneously engaging your abdominal muscles to lift your trunk to a 90° angle relative to the bed; and (e) swing both legs fully off the bed and settle into a sitting position.

Each task was repeated, up to three times, until each operator judged they had obtained a clear ultrasound clip. The acquisition process was conducted using the GE Healthcare Vivid iQ (© 2023 GE HealthCare, United Kingdom) ultrasound system, a portable and easy to carry device, equipped with a linear array transducer (3–9 MHz). This transducer had the capacity to scan to a depth of 40 mm below the surface of the skin. All the two-dimensional ultrasound clips were stored on the device’s hard drive for subsequent sternal micromotion data elaboration.

The ultrasound imaging acquisition process took place over two testing sessions: the first one occurred within 21 days after surgery, and the second one within three days following the first session. On day one, patients were evaluated by two different operators to assess inter-rater reliability. On day two, they were evaluated by only one of the operators to allow for the calculation of test–retest reliability from day one. In order to reduce order effect, the operators and the required task order were randomized by, respectively, a coin flip and a random sequence generation before the commencement of the session.

### 2.4. Off-Line Ultrasound Imaging Data Elaboration

The data elaboration process took place after the ultrasound imaging acquisition process. In this procedure, each operator had to evaluate every condition blindly, both from the other operators and from their own previous measurements, recording the data in independent sheets.

Each operator revised all his clips and selected the best one for each task. Sternal micromotions were then measured from the chosen clips using the built-in linear calliper function of the ultrasound software. In particular, sternal micromotion was recorded as the amount of separation or overlap of the sternal edges in the latero-lateral direction (coronal plane) and as the amount of separation in the antero-posterior direction (sagittal plane), measured in millimetres. Separation of sternal edges was denoted as a positive value, while overlap was denoted as a negative value. Regarding dynamic tasks, the operators were instructed to take the measurements describing the amount of separation/overlap at the point of the greatest displacement in relation to the starting position. They were allowed to take them using two different frames if the maximum displacement in the two directions took place in two different timings.

**Figure 1:**
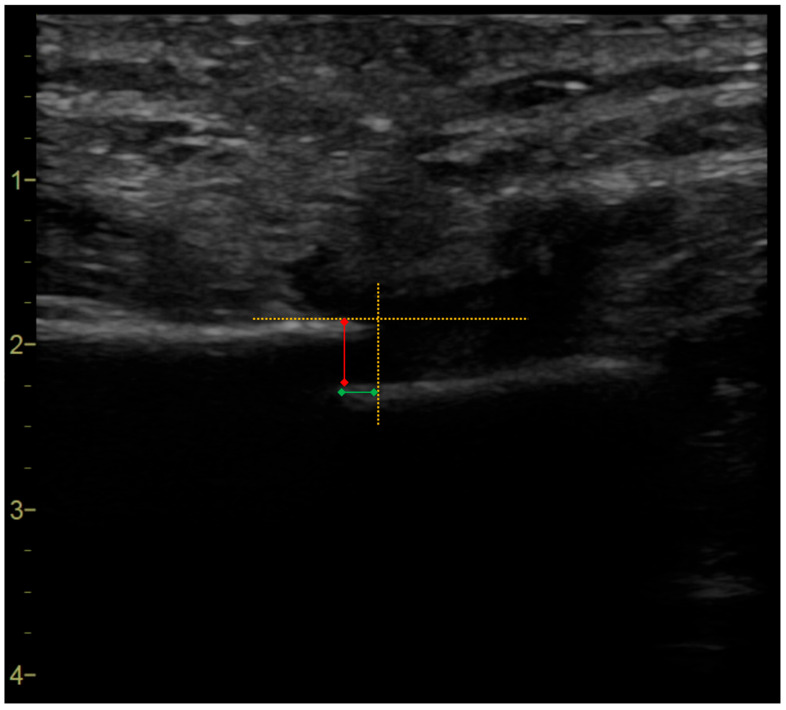
Example of an ultrasound frame. The figure shows part of the off-line ultrasound imaging data elaboration process, in which the operator who acquired the clip measures the distances between the sternal edges after selecting the frame that represents the maximum displacement in the sagittal and coronal planes (which, in this case, occur in the same frame). Using the built-in linear calliper function of the ultrasound software, the X- and Y-axes are traced to highlight the projections of the sternal halves (represented by the dashed orange lines), and the respective measurements are taken (the green and red lines).

Specifically, the two measurements obtained were as follows:

On the X axis, the distance between the medial margin of the upper sternal halve and the projection of the medial margin of the lower one on the line of the upper halve was measured, positioning the calliper as showed in the image (Figure 1):

On the Y axis, as the distance between the upper cortical margin of the upper sternal halve and the upper margin of the lower one (or its projection) was measured (see Figure 1).

The measurement procedure was conducted once for each task by the same operator who performed the ultrasound examination. Additionally, to assess intra-rater reliability, one of the two operators randomly performed the same measurement from the first day a second time, one day later.

As a result of this process, eight different measurements were obtained during each ultrasound evaluation, specifically assessing sternal separation or overlap along the following: (a) the X-axis: in the resting condition, during cough, during the rotational postural transition, and during the postural transition with IDSS; and (b) the Y-axis: in the resting condition, during cough, during the rotational postural transition, and during the postural transition with IDSS.

### 2.5. Statistical Analysis

Participant characteristics were presented as mean ± standard deviation (SD), whereas sternal displacement measurements along both axes were reported as median and Interquartile Range (IQR), expressed in millimetres.

Reliability was calculated for the sternal distance along both the X- and Y-axes, resulting in a total of eight measurements for each task. Test–retest and inter-rater reliability were assessed using intraclass correlation coefficient (ICC) (2, 1) absolute agreement, while intra-rater reliability was evaluated using ICC (3, 1) absolute agreement, each reported with their corresponding 95% confidence intervals (CI_95_) [21]. Reliability was classified as follows: ICC < 0.40 indicated poor reliability, 0.40–0.59 moderate reliability, 0.60–0.74 good reliability, and ≥0.75 excellent reliability [22].

The standard error of measurement (SEM), used to assess the absolute error of the instrument, and the minimum detectable change at the 90% confidence interval (MDC_90_), which represented the smallest change considered significant beyond the measurement error of an individual, were employed as measures of agreement [23]. The SEM was calculated by dividing the SD of the measurements by the square root of 1 minus the ICC (SD differences × √1-ICC). The MDC_90_ was derived using the following formula: 1.645 × √2 × SEM.

To assess the relationship between clinical instability and sternal displacement, Spearman’s rank correlation coefficient (ρ) was used. Specifically, correlations were tested between SIS scores and sternal displacement composite values, calculated for each patient as the mean difference between sternal distances measured during three dynamic tasks and at rest, separately for the antero-posterior and latero-lateral axes. The strength of the correlation was interpreted as follows: values of |ρ| < 0.30 were considered weak, 0.30–0.49 moderate, and ≥0.50 strong [24]. A *p*-value < 0.05 was considered statistically significant.

Statistical analyses were performed using R version 4.4.2 (R Core Team, 2024, Vienna, Austria) for Windows.

### 2.6. Sample Size Estimation

We assessed the inter-rater reliability between two trained physiotherapists, with rater 1 and rater 2 collecting data independently. It was hypothesized that the reliability between these raters would be excellent, defined as ICC (2, 1) ≥ 0.75. Sample size requirements for this reliability study were calculated based on the ICC method described by Bonett [25]. Assuming a reliability of approximately 0.8 between the two assessors using ultrasound imaging, and accounting for a dropout rate, a sample size of 35 patients was deemed sufficient to detect excellent reliability.

## 3. Results

The 35 patients with median sternotomy were consecutively enrolled and assessed at their bedside in the cardiologic rehabilitation ward of the ICS Maugeri. Assessments were conducted, on average, 15 (±4) days after the surgical intervention. Two patients were excluded because they declined to participate in the second evaluative session, which was required for the test–retest calculation. The characteristics of the study participants, including the presence or absence of various risk factors for sternal instability, are summarized in Table 1. Sternal instability, as assessed by the SIS, was clinically observed in only five patients. Among them, four patients had a SIS score of 1 (minimally separated sternum), while one patient had a score of 2 (partially separated sternum).

Table 2 showed the distances between the two sternal edges; in each condition, the median distances along the X-axis were consistently negative, reflecting an overlap of the sternal edges, while the Y-axis medians were positive, indicating a separation of the sternal edges.

**Figure 2:**
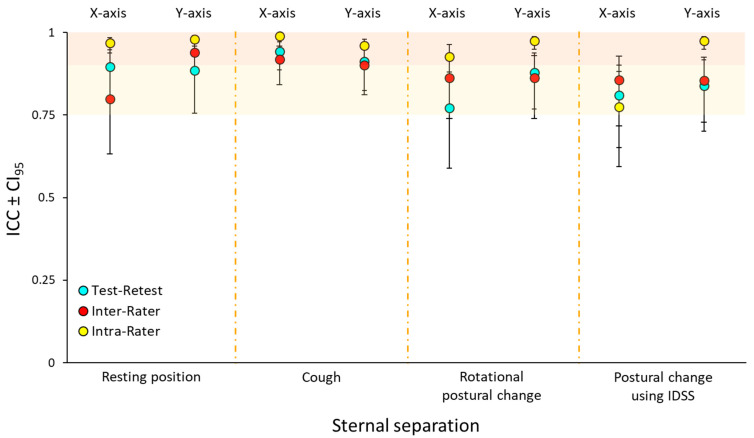
Reliability types across planes and movement conditions. Forest plot showing the three different reliabilities (light-blue, red, and yellow circles) with associated confidence intervals (CI_95_) for the four conditions, shown separately for antero-posterior (X-axis) and latero-lateral measurements (Y-axis). Shaded areas represent thresholds for excellent reliability (light shade ICC > 0.75; dark shade ICC > 0.90).

Test–retest and inter- and intra-rater reliability values are presented, separately for antero-posterior and latero-lateral measurements, in Figure 2. All ICCs were above 0.75, with intra-rater reliability demonstrating excellent values exceeding 0.90, except for the X-axis of postural change using IDSS.

Analyzing the test–retest (Table 3), the ICC values for the different measurements ranged from 0.77 to 0.94, indicating excellent reliability. Specifically, the resting position measurements showed high reliability, with ICCs of 0.90 for the X-axis and 0.89 for the Y-axis, both accompanied by relatively low SEMs (0.33 mm and 0.27 mm, respectively) and MDC_90_ values (0.78 mm and 0.62 mm, respectively). Similarly, during cough, the reliability was excellent, with ICCs of 0.94 for the X-axis and 0.91 for the Y-axis, with SEM and MDC_90_ values of 0.48 mm and 1.10 mm for the X-axis and 0.23 mm and 0.54 mm for the Y-axis. For rotational postural changes, the ICCs varied more noticeably between axes, with the X-axis presenting a reliability value of 0.77 and an excellent reliability of 0.88 for the Y-axis. SEM and MDC_90_ followed this trend, with the X-axis values of 0.60 mm and 1.40 mm, respectively, for SEM and MDC_90_, and the Y-axis values of 0.30 mm and 0.70 mm. Finally, postural changes measured using IDSS showed an ICC of 0.81 for the X-axis and 0.84 for the Y-axis. However, SEM and MDC_90_ values of 0.64 mm and 1.50 mm, respectively, were reported for the X-axis, while the Y-axis had values of 0.36 mm and 0.85 mm.

In addition, a moderate and statistically significant correlation was also found between latero-lateral displacement and SIS scores (ρ = 0.42, *p* < 0.05). This correlation was consistent across the three SIS levels.

## 4. Discussion

This study confirmed that ultrasound is a highly reliable tool for assessing sternal micromotions in post-sternotomy patients, with excellent intra-rater, inter-rater, and test–retest reliability. Physiotherapists with minimal ultrasound training were able to perform accurate and consistent measurements.

Among the three types of reliability, intra-rater reliability unsurprisingly achieved the highest values. This finding is likely due to our methodology, supported by other studies on intra-rater reliability [10], in which the same operator reviewed and remeasured the same video after a time interval, thereby eliminating the variability associated with repeated ultrasound imaging acquisition. Our results suggested that the variability of the off-line measurement process was minimal. In fact, reliability was generally higher when analyzing the same video twice rather than re-sampling and reassessing the same movement twice [26]. This result highlighted the need for caution when relying solely on this type of reliability in the context of ultrasound techniques [13].

When considering inter-rater reliability, the ICC values decreased. As expected, in our study, inter-rater reliability values were lower than intra-rater reliability values. Comparing our data with previous studies on inter-rater reliability is challenging, as in those studies, only the imaging data elaboration process was performed by two operators [10,11]. On the contrary, in our study, the entire procedure—i.e., image acquisition and data processing—was repeated by each operator involved in the inter-rater assessment. While this approach undoubtedly increased measurement variability, it was preferable, as it preserved a strong connection to real-world clinical practice, enhancing applicability. Our findings suggest that a proper standardization of image acquisition and measurement processes is sufficient to achieve good reliability among different operators [27,28].

Overall, the magnitude of all our reliability measures was excellent and comparable to those observed in other contexts, such as hyoid bone displacement evaluation, knee osteoarthritis, and tendon and muscle imaging [28,29,30,31,32]. This finding has significant implications for improving postoperative patient management. By enabling physiotherapists to quickly detect changes in a patient’s clinical condition, it allowed for timely and effective referrals to a cardiologist in cases of excessive variations or abnormalities in sternal stability [6,33]. Our data also suggested that, following a brief 10 h of training, physiotherapists with no prior experience in ultrasound evaluation were able to accurately assess sternal micromotions [34]. This finding is further supported by other studies, which suggest that physiotherapists with minimal training can perform real-time ultrasound imaging for shoulder joint translations or muscle evaluation with reliability levels comparable to those of experienced sonographers [35,36].

From a clinical standpoint, SEM and MDC values are essential for accurately interpreting the significance of ultrasound measurement results [23]. Under resting conditions, our SEM values for one plane of movement were quite similar to those of its respective orthogonal plane. However, they differed under dynamic conditions. Specifically, the indices for the X-axis nearly doubled those for the Y-axis, indicating greater measurement variability along the X-axis. This trend was also observed in the MDC_90_ values. These findings are consistent with existing literature. For instance, Pengelly et al. [11] reported that the greatest sternal micromotion during upper limb resistance exercises occurred in the X-axis, particularly during bicep curls. Moreover, inter-rater reliability for lateral measurements was lower (ICC = 0.73) compared to the antero-posterior direction (ICC = 0.83), highlighting the inherent difficulty in capturing latero-lateral sternal motion with ultrasound. These results are superimposable to our findings of lower ICC and higher SEM/MDC values along the X-axis, suggesting that the ultrasound assessment of lateral displacement remains more technically challenging. Also, our physiotherapists agreed that measurements along the X-axis were generally more difficult than those along the Y-axis. Specifically, they noted greater difficulty in identifying the medial cortical margins of the sternal edges, which often appeared blurred and less sharply defined. In contrast, the superior margins were consistently clearer under all conditions. This discrepancy was likely due to motion artefacts caused by patient movement, which compromised the stability of the ultrasound probe, further complicating the identification of a healing bone stump that had been cut during surgery. While advanced solutions such as speckle-tracking or 3D ultrasound may help improve the precision of lateral sternal micromotion measurements, their routine clinical use remains limited by current availability and cost. Speckle-tracking may be more readily implementable through software enhancements, whereas 3D ultrasound, though promising, is still confined to specialized settings [37,38]. Additionally, semi-automated measurement algorithms and standardized patient positioning protocols may further improve their consistency.

Despite these issues, our study confirms the ability of ultrasound to detect micromovements below 2 mm, as all of the MDC_90_ values we calculated were below this threshold. This cut-off has been previously proposed for evaluating the safety of movements [11,14], as the literature has suggested that micromovements of the sternal edges exceeding 2 mm could lead to necrosis and impair bone healing [39]. Our findings suggest that the identified threshold is particularly meaningful for latero-lateral measurements, which were the only ones to approach or exceed it. Antero-posterior displacements remained consistently below 2 mm, even in clinically unstable patients. This supports the use of latero-lateral ultrasound assessments as a more sensitive indicator of potentially harmful shear forces on the healing sternum.

Therefore, ultrasound imaging may be a valuable tool for enhancing post-surgery monitoring and management, aiding in surgical decision-making and identifying dangerous exercises and activities, even those involving complex trunk movements like the postural transitions in our study [11,40,41]. As a matter of fact, this approach may be particularly valuable for patients who report discomfort, show signs of suspected instability during functional tasks such as the supine-to-sitting transition, or have several risk factors. In such scenarios, displacement values exceeding the MDC_90_, especially those nearing the 2 mm threshold reported to hinder bone healing [39], could serve as clinical red flags, warranting closer monitoring. Although further validation is required, this method has the potential to inform exercise prescription and guide individualized rehabilitation planning. Future studies should further investigate threshold-based decision-making in larger, more heterogeneous cohorts.

The testing procedure, lasting approximately 30 min, was non-invasive and well tolerated by the patients, with no apparent adverse events. Additionally, ultrasound is a cost-effective tool, capable of providing real-time feedback on sternal micromotion in a reliable way, and widely used in emergency settings [42].

Our article had several potential limitations. One of them lies in the limited number of movements we investigated. Unlike previous studies, we have not considered many movements beyond postural transitions, such as unilateral and asymmetric upper limb movements or the use of isotonic machines, commonly employed in postoperative rehabilitation [43,44]. Another limitation of this study was the absence of a criterion validity analysis against a gold-standard imaging modality such as radiostereometric analysis, computed tomography, or intraoperative measurements [45]. Although our findings supported the reliability of ultrasound-based assessments, further studies are needed to confirm the accuracy of this method by comparing it with established reference techniques in both experimental and clinical settings. Additionally, the low percentage of women in our sample (15%) limited the generalizability of our findings. Given that older women are more prone to postoperative complications due to factors such as osteoporosis and macromastia [46,47], future studies should investigate whether these conditions influence the reliability of ultrasound measurements of sternal micromotion. Lastly, only 15% of our participants showed clinically detectable sternal instability according to the SIS. Indeed, our primary objective was to assess the overall reliability of ultrasound-based measurement procedures when performed by physiotherapists, independently from their stability condition. The inclusion of mostly stable patients may have limited the applicability of our results to populations with greater instability. Nevertheless, establishing baseline measurement consistency in a general postoperative cohort is a necessary first step before extending the method to higher risk cases. It is important to note that ICC is sensitive to between-subject variance [48]. In our relatively homogeneous sample, where most patients exhibited low sternal instability, this variance was limited, potentially leading to conservative ICC estimates. Therefore, the excellent reliability observed may actually have underestimated the true robustness of the method, which could become even more evident in a more heterogeneous population.

## 5. Conclusions

Ultrasound performed by physiotherapists with no prior experience has proven to be a reliable tool for measuring sternal micromotion in post-sternotomy patients. A 10 h training programme was sufficient to ensure accurate and consistent measurements, suggesting that prior experience did not significantly affect measurement precision. Our study was designed with a high level of ecological validity, making it more reflective of real-world clinical practice compared to previous research. The quantitative assessment of sternal micromotions via ultrasound could be a valuable addition to clinical practice for detecting, measuring, and monitoring sternal instability. It also had the potential to serve as an outcome measure to guide treatment decisions and contribute to redefining sternal precautions in rehabilitation [49]. Given its reliability, practicality, and low cost, the use of ultrasound by physiotherapists should be encouraged.

## Figures and Tables

**Table 1:** Characteristics of participants (N = 33).

N = 33	Mean ± SD
Age (yrs)	65.9 ± 7.1
BMI (kg/m^2^)	25.0 ± 3.5
Respiratory rate (number/min)	19.7 ± 3.4
Thorax circumference (cm)	99.8 ± 10.3
	**Frequency**
Sex (M)	28 (85%)
Diabetes	8 (24%)
COPD	2 (6%)
Smoking history	17 (52%)
Osteoporosis	1 (3%)
Bilateral mamillary artery usage	3 (9%)
Sternal instability by SIS	5 (15%)

SD, standard deviation; BMI, body mass index; M, male; COPD, chronic obstructive pulmonary disease; SIS, sternal instability scale.

**Table 2:** Distances of sternal edges along the X- and Y-axes across different conditions.

N = 33	X-Axis (mm)			Y-Axis (mm)		
	Median	IQR1	IQR3	Median	IQR1	IQR3
Resting position	−1.6	−2.3	−1.2	1.3	0.8	1.7
Cough	−0.9	−1.7	−0.6	1.2	0.6	1.6
Rotational postural change	−0.8	−1.4	−0.3	1.3	0.7	1.7
Postural change using IDSS	−0.9	−1.3	0.3	1.4	0.8	2.0

IDSS, individual device for supine-to-sitting; IQR1, First Interquartile Range; IQR3, Third Interquartile Range.

**Table 3:** Test–retest reliability of ultrasound in measuring sternal micromotion.

Variable	ICC	CI_95_		SEM (mm)	MDC_90_ (mm)
		Lower	Upper		
Resting position, X-axis	0.90	0.80	0.95	0.33	0.78
Resting position, Y-axis	0.89	0.76	0.94	0.27	0.62
Cough, X-axis	0.94	0.89	0.97	0.48	1.10
Cough, Y-axis	0.91	0.82	0.96	0.23	0.54
Rotational postural change, X-axis	0.77	0.59	0.88	0.60	1.40
Rotational postural change, Y-axis	0.88	0.77	0.94	0.30	0.70
Postural change using IDSS, X-axis	0.81	0.65	0.90	0.64	1.50
Postural change using IDSS, Y-axis	0.84	0.70	0.92	0.36	0.85

CI_95_, 95% Confidence Interval; ICC, Intraclass Correlation Coefficient; MDC_90_, 90% Minimal Detectable Change; SEM, Standard Error of Measurement.

## Data Availability

The data that support the findings of this study are available from the corresponding author, IA, upon reasonable request.
